# Advances in Metamaterials: Structure, Properties and Applications

**DOI:** 10.3390/ma19010085

**Published:** 2025-12-25

**Authors:** Bin Zheng, Peixuan Zhu

**Affiliations:** State Key Laboratory of Extreme Photonics and Instrumentation, ZJU-Hangzhou Global Scientific and Technological Innovation Center, Zhejiang University, Hangzhou 310027, China

Metamaterials, leveraging the unique characteristics of subwavelength structures, have led to significant research progress in fields such as acoustics, optics, and electromagnetics [1,2,3,4]. By enabling precise control of topological morphology and component arrangement at the microscale, these materials exhibit functional properties beyond those of conventional materials, achieving novel functionalities such as sound field manipulation, non-reciprocal transmission, and highly sensitive sensing [5,6,7,8]. From noise control to intelligent communications, and from invisibility technology to biosensing, metamaterials are playing an increasingly important role in bridging fundamental research and engineering applications. This Special Issue (SI), “Advances in Metamaterials: Structure, Properties and Applications”, focuses on the latest advances in theoretical, experimental, and simulation research in this field, and this article provides an overview of the 14 research papers collected in this SI.

In the field of acoustic metamaterials research, noise suppression and sound field control represent particularly important and widely studied topics [9,10,11,12,13]. To address the challenge of high-dimensional non-convex optimization in the design of diffuse-reflecting metamaterials, Ma et al. proposed a hybrid algorithm (VDGD) combining the tornado algorithm and the gradient descent method [11]. Using a two-stage strategy of global perturbation and local fine-tuning, they significantly reduced the standard deviation of the sound field from 5.81 dB to 1.91 dB, demonstrating notable optimization effectiveness. By optimizing stackable and expandable multi-tortuous channel acoustic metamaterials (SEAM-MTCs), Bi et al. achieved a substantial increase in the average sound absorption coefficient across the 200–6000 Hz frequency band, offering a practical approach for noise reduction in large mechanical equipment [12]. Further optimizing the aperture shape of Helmholtz resonators (HRs), Bi et al. found that resonators with an olive-shaped tangential cross-section and a circular transverse cross-section exhibit lower resonance frequencies, providing key insights for the miniaturized design of low-frequency sound-absorbing devices [13].

Recent advances in optical and electromagnetic metamaterials have demonstrated significant breakthroughs in non-reciprocal transmission, multi-functional reconfiguration, broadband absorption, and simulation efficiency, thereby contributing to the development of high-performance optoelectronic devices [14,15,16,17,18,19,20,21]. Wu et al. integrated highly nonlinear liquid metamaterials (LMMs) with two-dimensional silicon dielectric gratings to create a novel non-reciprocal electromagnetic metamaterial, achieving a forward-to-backward transmission coefficient contrast ratio as high as 0.96 while significantly reducing the radiation power required for a nonlinear response [17]. Leveraging the phase-change properties of vanadium dioxide (VO_2_), Shan et al. developed a polarization-insensitive, multi-functional reconfigurable metamaterial capable of flexibly switching between electromagnetically induced absorption (EIA), electromagnetically induced transparency (EIT), and asymmetric absorption modes, while simultaneously enabling the photonic spin Hall effect, spin-selective absorption, and beam deflection [18,19]. This finding opens a new path for the multi-functional integration of terahertz devices. Kim et al. employed a genetic algorithm (GA) to optimize the combination of copper sheets and chip resistors, designing a broadband metasurface absorber that provides a low-cost design strategy for highly efficient microwave-absorbing devices [20]. Regarding simulation efficiency, Wang et al. accounted for electromagnetic coupling between meta-atoms and reduced the simulation deviation of metal lenses and beam splitters by 97% compared to conventional methods, removing a major efficiency barrier to large-scale metamaterial design [21].

Intelligence and integration are emerging as central trends in metamaterial development, where the fusion of deep learning and adaptive systems is accelerating functional advancements and practical applications [22,23,24,25,26,27]. To address the low efficiency in predicting the spectra of high-Q dielectric metamaterials, Liao et al. utilized transfer learning to achieve accurate prediction of near-infrared transmission spectra, providing an efficient tool for metamaterial design [25]. Lu et al. combined the soft actor–critic algorithm with reconfigurable metamaterials to achieve adaptive beam focusing at arbitrary locations in obstructed environments, offering an innovative solution to the path loss problem for blocked users in 6G communications [26]. Li et al. developed an intelligent metamaterial cloak system integrated with sensing units, which uses an FPGA to perceive the incident wave direction and frequency in real time and employs adaptive feedback control to reconfigure the reflection phase. As a result, the system achieves multi-directional invisibility without manual intervention, overcoming the directional limitations of traditional cloaking devices [27].

Innovations in structural design and fabrication processes have laid a crucial foundation for performance optimization and scalable applications of metamaterials [28,29,30,31,32,33]. Based on triply periodic minimal surface (TPMS) sandwich structures, Vasile et al. used implicit modeling methods to generate eight shell-based topology types and one random structure, offering new design concepts for lightweight, high-strength structural components [31]. Straub et al. used laser powder bed fusion to fabricate NiTi filament structures that exhibited exceptional superelasticity, with the critical stress for martensitic transformation being controllable through process parameters, enabling tailored micro-properties for programmable metamaterials [32]. Qiu et al. combined Fabry–Pérot cavity (FPC) theory with a phase-gradient partial-reflection metamaterial (PGPRM) fishnet structure, achieving a 29° beam deflection and a maximum gain of 16.9 dBi in the 8.6–9.2 GHz band, providing a low-cost solution for signal enhancement and redirection in indoor wireless communications [33].

The development of metamaterials is entering a golden age, marked by multidisciplinary integration, intelligence-driven innovation, and accelerated practical applications [34,35,36]. In the future, through deeper integration of artificial intelligence and advanced manufacturing technologies, metamaterials are expected to achieve further breakthroughs in design efficiency, functional integration, and environmental adaptability. From multi-scale optimization of microstructures to multi-functional integration across frequency bands and scenarios [37,38], and from laboratory prototypes to mass-production applications [28,29], metamaterials will undoubtedly play increasingly critical roles in intelligent devices [22,23,39,40], communication technologies [41,42], and other fields, injecting sustained momentum into scientific and technological progress in human society.
